# Selenium Intake in Iodine-Deficient Pregnant and Breastfeeding Women in New Zealand

**DOI:** 10.3390/nu11010069

**Published:** 2019-01-01

**Authors:** Ying Jin, Jane Coad, Janet L Weber, Jasmine S Thomson, Louise Brough

**Affiliations:** 1 School of Health Sciences, College of Health, Massey University, Palmerston North 4442, New Zealand; y.jin@massey.ac.nz; 2 School of Food and Advanced Technology, College of Sciences, Massey University, Palmerston North 4442, New Zealand; j.coad@massey.ac.nz (J.C.); j.l.weber@massey.ac.nz (J.L.W.); j.a.thomson@massey.ac.nz (J.S.T.)

**Keywords:** selenium, pregnancy, lactation, breastfeeding, infants

## Abstract

Selenium plays a role in antioxidant status and, together with iodine, in thyroid function. Iodine deficiency exists in New Zealand during pregnancy and lactation, and selenium deficiency may further affect thyroid function. This study investigated selenium intakes of pregnant and lactating women, in Palmerston North, in the North Island of New Zealand. Dietary intake was estimated using three repeated 24-h dietary recalls. Dietary intake in pregnancy was also estimated from 24-h urinary excretion of selenium. Selenium concentrations were determined in urine and breastmilk using inductively-coupled plasma mass spectrometry. Median selenium intakes based on dietary data were 51 (39, 65) μg/day in pregnancy and 51 (36, 80) μg/day in lactation, with 61% and 68% below estimated average requirement (EAR). Median daily selenium intake in pregnancy based on urinary excretion was 49 (40, 60) µg/day, with 59% below EAR. Median selenium concentration in breastmilk was 11 (10, 13) µg/L and estimated median selenium intake for infants was 9 (8, 10) µg/day, with 91% below the Adequate Intake of 12 μg/day. These pregnant and breastfeeding women were at risk of dietary selenium inadequacy. Further research is required to assess selenium status in relation to thyroid function and health in this group.

## 1. Introduction

The intake of selenium worldwide ranges from 7 to 4990 µg/day, and varies greatly from deficient to toxic intakes [1]. New Zealand soils contain low levels of selenium, leading to low levels in the food supply [2]. The most recent New Zealand Total Diet Survey suggested dietary selenium intake was inadequate throughout the New Zealand population, putting them at risk of deficiency [3]. Recent New Zealand studies have shown low selenium intakes in women of childbearing age and older women based on urinary selenium excretion [4,5].

Selenium is essential in human health to produce selenoproteins, which have antioxidant and anti-inflammatory roles, and also for production of thyroid hormones [6]. Selenoproteins (iodothyronine deiodinases) are required for generating the active thyroid hormone T_3_ (triiodothyronine) from the inactive T_4_ (thyroxine) form [7]. Selenium is also an essential cofactor for glutathione peroxidase, a potent antioxidant, which protects thyroid cells from damage due to any excessive hydrogen peroxide generated from the synthesis of thyroid hormones [8].

Selenium has been suggested to play an important role in normal brain development, although the mechanism is not clear. Two recent large cohort studies from Poland and Spain found selenium status in first trimester was adversely associated with neuropsychological development assessed at 1 year and 2 years of age by the Bayley Scales of infants and Toddler development [9], and 5 years of age by the McCarthy Scales for Children’s Abilities (MSCA) [10]. Varsi et al. (2017) investigated the effect of maternal selenium status on neurodevelopment of infants and reported that low serum selenium concentration in pregnancy was negatively associated with infant psychomotor score at 6 months of age [11].

The interaction between selenium and iodine in thyroid hormone synthesis is of particular concern in New Zealand due to dietary insufficiency of both selenium and iodine. Iodine deficiency has historically been a health problem in New Zealand [12] and the mandatory fortification of all bread (except organic) with iodised salt was introduced in September 2009 [13]. Since mandatory fortification, the majority of adults [14] and school-aged children [15] in New Zealand have adequate iodine intakes. Despite an iodine supplement being recommended and available to all pregnant and lactating women in New Zealand, this population group still has insufficient intakes and low status [16]. Selenium deficiency could potentially exacerbate the consequences of mild iodine deficiency among this vulnerable group [12].

During pregnancy and lactation, there are increased selenium requirements for the growing foetus and newborn [3]. Low maternal serum selenium concentrations are associated with adverse pregnancy outcomes such as pre-eclampsia [17], other types of pregnancy-induced hypertension [18] and preterm birth [19]. Human milk is critical for an exclusively breastfed infant’s optimal selenium status. A study in the South Island of New Zealand (1998–1999) showed postpartum women and breastfed infants had low plasma selenium, suggesting suboptimal status [20]. Since then, no data about selenium intakes have been collected for this population. Given changes in dietary habits, food product availability and agricultural practices, continual monitoring of selenium intake in this vulnerable population is essential.

This study aimed to assess current maternal selenium intake during pregnancy and lactation, and estimate infant selenium intake in a sample of women in Palmerston North, North Island, New Zealand.

## 2. Materials and Methods

### 2.1. Study Population

Pregnant and breastfeeding women were recruited from January to July 2009 and January to September 2011 via local health professionals who work closely with pregnant and breastfeeding women, as described previously [16]. Volunteers were aged 16 years and older, in their third trimester of pregnancy (greater than 26 weeks of gestation), or at least 3 weeks postpartum and breastfeeding. Women who had medical complications during their pregnancy were excluded. In total, 59 pregnant and 68 lactating women were recruited and included in the study. Women had to actively volunteer for this study and no data were kept from women who did not meet the selection criteria.

Ethical approval was obtained from the Massey University Human Ethics Committee (Southern A 08/32 and 10/54). Written consent was obtained from all participants.

### 2.2. Dietary Data Collection

A 24-h dietary recall was conducted based on the US Department of Agriculture Automated Multiple-Pass Method, but excluded the Forgotten Foods List [21]. A photographic food atlas was provided to estimate portion sizes [22]. Participants were also asked to include any dietary supplements taken, including the brand name and the amount. Two subsequent recalls were collected via telephone interviews over the following two weeks, ensuring a weekend day was included; food portion sizes were estimated using household measures. Previous research has found no difference in energy intakes when comparing 24-h dietary recalls collected in person versus via the telephone [23]. Dietary data were analysed using Foodworks 2009 (Xyris Software, Brisbane, Australia) based on the New Zealand food database. Dietary supplements used by participants were included in dietary data analysis. Only 4 of the 59 pregnant and 6 of the 68 lactating women were taking selenium-containing supplements.

The estimated average requirement (EAR) cut-point method can be used to assess population nutrient intake providing nutrient requirements are normally distributed (e.g., selenium); the percentage below the EAR approximates the proportion that is at risk of dietary inadequacy [24]. For a population to have a very low prevalence of inadequate dietary intakes, the mean/median intake should be above the recommended daily intake (RDI) [24]. Current intakes based on diet and urine data were compared to Australian and New Zealand recommendations; the Estimated Average Requirement (EAR) and Recommended Dietary Intake (RDI) for selenium for pregnant women are 55 and 65 µg/day, and for lactating women are 65 and 75 µg/day, respectively [3].

### 2.3. Sample Collection and Selenium Analysis of Urine and Breastmilk

All participants were asked to collect a 24-h urine sample and provided with an insulated box containing two polythene bottles for urine storage and frozen silica pads to keep the sample cool. Lactating women were also requested to provide a breastmilk sample (around 30 mL) and provided with a breast pump if required; timing of collection of breastmilk samples was not standardized, since no significant differences have been found in selenium concentrations between hind-milk and fore-milk [25]. The concentration of selenium in breastmilk varies most significantly during the first 21 days from the transition from colostrum to mature milk [25], thus breastmilk samples were collected after 3 weeks postpartum. All samples were brought immediately to the Human Nutrition Research Unit for processing after collection. The total volume of urine collected over 24 h was measured for each participant. Samples were stored without preservative at −20 °C, prior to analysis. Urine samples were defined as inaccurate if urine volume was below 1 L and urinary creatinine below 5 mmol/day, or extreme outliers of creatinine (>3 Standard Deviation) [26]. However, no study samples were classified accordingly.

Selenium concentrations of urine and breastmilk samples were determined by Hill Laboratories, Hamilton, New Zealand, using inductively-coupled plasma mass spectrometry [27]. Quality Control procedures included analysis of blanks, analytical repeats and spiked samples in order to ensure accuracy and precision. Calibration standards and checks were undertaken on every run with the limit of detection at 0.002 mg/kg. Dietary selenium intake was estimated for pregnant women, based on a urinary excretion of 55% of selenium intake [28]. However, it was not possible to estimate dietary selenium intake for lactating women via urine, as we were unable to determine the daily loss of selenium from breastmilk. Creatinine was measured using the Jaffe Method Flexor E (Vital Scientific NV, 6956 AV Spankeren/Dieren, Rheden, Gelderland, The Netherlands) at Massey University Nutrition Laboratory.

### 2.4. Statistical Analysis

Data were analysed using IBM SPSS (Statistics Package for the Social Sciences, IBM, Armonk, NY, USA) version 20. Data were tested for normality using Shapiro-Wilk’s test. Non-parametric data were expressed as median (Quartile 1, 3 (Q1, Q3); based on weighted average) and parametric data expressed as mean (±standard deviation; SD). Bivariate correlations were tested using the nonparametric Spearman’s rho correlation coefficient. Scatter plots were generated for suspected bivariate correlations and visually inspected for verification. Fisher’s exact test was used to detect associations between dietary and biological methods in assessing dietary intake.

## 3. Results

Fifty-nine pregnant and 68 lactating women were recruited. The mean age was 31.6 ± 5.7 and 31.3 ± 5.0 years for pregnant and breastfeeding women, respectively (Table 1). The ethnicities of participants were Caucasian (80%, 81%), Maori (12%, 9%), Asian (5%, 2%) and other (3%, 8%). Participants were predominantly educated at tertiary level (86% pregnant and 68% breastfeeding), with approximately half being pregnant with or breastfeeding their first infant.

Median urinary selenium for pregnant women was 14.1 (9.1, 18.2) µg/L (Table 2) and median selenium intake based on urinary excretion was 49 (40, 60) µg/day (Table 3), below both the RDI (65 µg/day) and EAR (55 µg/day), with 59% below the EAR. Median selenium intake based on dietary assessment among pregnant women was 51 (39, 65) µg/day, below both the RDI and EAR, with 61% below the EAR (Table 3). Urinary and dietary data both suggest inadequate selenium intakes among pregnant participants.

Based on dietary assessment, the median selenium intake for breastfeeding women was 51 (36, 80) µg/day (Table 3), also below both the EAR (65 µg/day) and RDI (75 µg/day), with 68% below the EAR. Median selenium concentration in breastmilk (n = 64) was 11 (10, 13) µg/L (Table 2). Using an estimated daily breastmilk intake of 750 mL [29], the median estimated selenium intake for infants was 9 (8, 10) µg/day; 70% (45/64) were below the daily minimum of 10 µg/day suggested by Levander [30], and 91% (58/64) below the Adequate Intake of 12 µg/day [31].

For breastfeeding women, selenium concentration in breastmilk was weakly positively correlated with 24-h selenium excretion in urine as µg/day (*p* = 0.269, *r* = 0.032, see Table A2). Pregnant participants’ dietary selenium intake based on dietary assessment was not associated with selenium excretion as either µg/L (*p* = 0.053, *r* = 0.692) or µg/day (*p* = 0.230, *r* = 0.079; see Table A1). However, the classifications of intakes as either above or below the EAR were associated for the two methods of assessing dietary intake (*p* = 0.016, Fisher’s exact test).

## 4. Discussion

This study found 59–61% of pregnant and 68% breastfeeding participants had estimated selenium intakes below the EAR, suggesting this vulnerable group is at risk of an inadequate selenium intake. This supports the latest New Zealand Adult Nutrition Survey 2008/2009, which estimated that 44–72% of women aged 19–50 years had inadequate selenium intakes [32]. Previous research shows that low selenium status is associated with an increased risk of thyroid enlargement, which may indicate compromised thyroid function [33]. Iodine deficiency has previously been reported in both pregnant and breastfeeding women in New Zealand in the same cohort investigated in this study [16], and selenium deficiency could further compromise thyroid function.

In the present study, dietary intake was assessed by three 24-h dietary recalls, due to its low participant burden and good compliance. Under- or over-reporting is a concern for dietary assessment. As energy expenditure was not recorded, we were unable to determine if participants had misreported dietary intake. A large daily variation of selenium intake was reported in an earlier study of American pregnant and postpartum women using duplicate-plate food and drink composites and dietary recalls [34]. Single 24-h recalls do not take into account day-to-day variation, therefore repeated 24-h dietary recalls are frequently used to estimate usual intake [35].

In the current study, 24-h urinary selenium excretion was used to estimate selenium intake. It is estimated that 50–60% of dietary selenium is excreted in urine [28], and selenium intake determined in this manner is suggested to be more accurate than dietary assessment data [36]. However, collecting 24-h urine samples requires motivated participants and is not practical for all populations or large studies. Urinary selenium has been shown to be a valid method to assess recent selenium intake in populations that live in selenium-deficient areas [36,37]. Research has shown that serum selenium and glomerular filtration rate increase in pregnancy, and studies have shown an increase in selenium in urine during pregnancy [38]. Thus, the selenium excretion of 55% could be overestimated, so actual selenium intakes could be even lower than estimated values. A previous New Zealand study found selenium intake determined from a Food Frequency Questionnaire was associated with 24-h urine excretion in pregnant women [38]. Although the current study found no such association in pregnant women, the classification of intakes as either above or below the EAR was associated for the two methods of assessing dietary intake.

Median intake of selenium for pregnant women in the current study was 51 µg/day based on dietary intake and 49 µg/day based on urine excretion. In previous studies of New Zealand pregnant women, Watson and McDonald found median intakes ranging between 33.5 µg/day excluding dietary supplements to 67 mcg/day including dietary supplements [39], however, these data were based on dietary assessment with no verification using biomarkers. The median selenium intake of 51 µg/day for breastfeeding women was higher than previously reported (46 µg/day) in the 1998–1999 study of lactating mothers from the South Island of New Zealand [20]. This was not unexpected, as selenium intake is typically lower in the South Island of New Zealand, where bread is made from local wheat, compared to the North Island, where bread is manufactured from wheat imported from Australia, which has higher levels of soil selenium [12]. It could also be due to changes occurring in diet in the last 20 years. Even though selenium intake is higher among breastfeeding women in the current study than previously reported, many current intakes are still below the EAR, thus suggesting a risk of dietary inadequacy.

Breastmilk selenium concentration is associated with maternal selenium intake and/or status. Selenium is generally higher in colostrum (26 µg/L), and then decreases to nadir levels in mature milk (1–3 months, 15 µg/L) [30]. Median selenium breastmilk concentrations (11.3 µg/L) in the present study were similar to those reported in the South Island in 1992 (13.4 µg/L) [40] and also a recent study in the North Island (14 µg/L) [41]. Adequate selenium concentrations in breastmilk have been observed to maintain optimum selenium status in both preterm and term infants [25]. For exclusively breastfed infants, breastmilk is the only source of selenium; in the current study, 70% of infants would not have achieved the 10 µg/day suggested as adequate by extrapolation from adults [30] and 91% did not achieve the Adequate Intake of 12 µg/day [18]. This suggests infants in the present study are at risk of selenium deficiency.

The inadequate selenium intakes in this vulnerable population are of concern. Studies in rats have previously shown that in utero selenium deficiency can impair neonatal lung development [42]. Maternal selenium status in French women was negatively associated with risk of wheezing in children aged 1–3 years; this could potentially lead to asthma later in life [43]. Low selenium status in childhood in New Zealand has also been associated with increased risk of wheeze [44], for which New Zealand has a high incidence [45]. Low maternal selenium status in Norwegian women has been associated with an increased risk of neonatal infections in the first 6 weeks of life and lower psychomotor score at 6 months [11]. Adequate dietary intake of selenium has been suggested to be beneficial in improving mental outlook among the general population [46]. Lower dietary selenium intake has also been associated with an increased risk of de novo major depressive disorder among women [47]. Selenium supplementation during early pregnancy has been found to reduce postnatal depression [48], which has a 7.8% to 16% prevalence in New Zealand [49].

Determining selenium concentrations in blood (whole, plasma or erythrocyte), plasma selenium protein P or GPx activity in blood (whole, plasma or platelet) are considered more reliable markers of selenium status [50,51]. However, urinary selenium excretion is associated with both plasma selenium and dietary intake in populations with low selenium intake [12]. A limitation of the current study is not measuring selenium or GPx activity in blood, however, determining daily urinary selenium excretion serves as a proxy measure for selenium intake and indicates the need for further research.

This study included a small sample of pregnant and breastfeeding women who were predominantly well educated and more likely to be affluent, thus the sample is not representative of the New Zealand population. However, women who volunteer for health studies tend to be interested in health and motivated towards a healthy lifestyle, thus it is of concern that these women are at risk of selenium deficiency. Further, we would not expect such women to have a poorer health status than less affluent women.

Additionally, supplement intakes could contribute to participants’ dietary selenium intake; however, only a small proportion of participants consumed selenium-containing supplements. Thus, we were not able to meaningfully investigate the potential impact of supplement intake on other measures.

## 5. Conclusions

This current research suggests dietary selenium intake is a concern for pregnant and breastfeeding women and their infants in New Zealand. Further research is required to assess selenium status among these groups by measuring biomarkers such as plasma selenium or GPx activity in blood selenium. Further investigations should also include all socio-economic groups. It is essential that we assess whether suboptimal intake of selenium adversely affects thyroid function in this already iodine-deficient population. As selenium is a nutrient with numerous roles, it is also necessary to investigate any effects of low intake on other health outcomes potentially related to selenium in the perinatal period, such as postnatal depression and infant neurodevelopment.

## Figures and Tables

**Table 1:** Description of pregnant and breastfeeding participants.

*n* (%)	Pregnant	Breastfeeding
	*n* = 59	*n* = 68
Age, years (Mean ± SD)	31.6 ± 5.7	31.3 ± 5.0
Tertiary Education	51 (86)	46 (68)
Ethnicity (Caucasian)	47 (80)	56 (81)
Ethnicity (Maori)	7 (12)	6 (9)
Ethnicity (Asian)	3 (5)	1 (2)
Ethnicity (Other)	2 (3)	5 (7)
Nulliparous	31 (53)	-
First time lactation	-	36 (53)
Gestational age, days (Median (Q1, Q3))	207 (191, 247)	
Age of infants, days (Mean ± SD)		113.4 ± 96.9

**Table 2:** Selenium and creatinine in 24-h urine samples from pregnant and breastfeeding women and selenium in breastmilk from breastfeeding women.

Median (Q1, Q3)	Pregnant	Breastfeeding
Numbers of participants (*n*)	59	68
Urine volume (L)	2.2 (1.5, 3.0)	1.8 (1.2, 2.5)
Urinary selenium concentration µg/L	14.1 (9.1, 18.2)	12.1 (7.8, 19.9)
Measured 24-h urinary selenium µg/day	27.1 (22.0, 32.9)	21.2 (14.5, 29.9)
Urinary creatinine g/L	0.5 (0.4, 0.7)	0.7 (0.5, 1.1)
Urinary creatinine g/day	1.2 (1.0, 1.5)	1.3 (1.2, 1.4)
Selenium: creatinine µg/g	22.8 (17.7, 28.7)	16.5 (12.3, 23.8)
Selenium in breastmilk µg/L	-	11.3 (10.0, 13.3) ^a^

^a^*n* = 64 for breastmilk samples.

**Table 3:** Estimated selenium intake in pregnant and breastfeeding women, infants and comparison to recommendations.

Selenium Intake	Pregnant	Breastfeeding	Infant
	(*n* = 59)	(*n* = 68)	(*n* = 64)
Estimated selenium intake; median (Q1, Q3)			
Based on 24-h urine, µg/day	49 (40, 60)		
Based on 24-h dietary recalls, µg/day	51 (39, 65)	51 (36, 80)	-
^a^ Below EAR (n, %)			
Based on 24-h urine	35 (59)		
Based on 24-h dietary recalls	36 (61)	45 (68)	-
Estimated selenium intake; median (Q1, Q3)			
Based on 750 mL breastmilk per day	-	-	9 (8, 10)
Below (10 µg/day) (n, %)	-	-	45 (70)
Below (12 µg/day) (n, %)	-	-	58 (91)

^a^ EAR = estimated average requirement, 55 µg/day for pregnant women and 65 µg/day for breastfeeding women.

## Appendix A

**Table A1:** Correlation matrix for selenium and creatinine in 24-h urine samples in pregnant women using Spearman’s rho.

*n* = 59		Urine	Urine Selenium	Estimated Selenium Intake (Urine)	Urine Creatinine	Urine Creatinine	Selenium: Creatinine Ratio	Estimated Selenium Intake (Dietary Data)
		Volume L	µg/L	µg/day	g/L	g/day	µg/g	μg/day
Urine Selenium µg/L	*r*	−0.719						
	*p*	0.000						
Estimated Selenium Intake (Urine) µg/day	*r*		0.485					
	*p*	ns	0.000					
Urine Creatinine g/L	*r*	−0.863	0.760					
	*p*	0.000	0.000	ns				
Urine Creatinine g/day	*r*			0.380	0.259			
	*p*	ns	ns	0.003	0.047			
Selenium Creatinine Ratio µg/g	*r*		0.466	0.804				
	*p*	ns	0.000	0.000	ns	ns		
Estimated Selenium Intake (Dietary Data) µg/day	*r*			0.230				
	*p*	ns	ns	0.079 (ns)	ns	ns	ns	
Total Energy Intake kJ	*r*							0.263
	*p*	ns	ns	ns	ns	ns	ns	0.044

ns = not statistically significant.

**Table A2:** Correlation matrix for selenium and creatinine in 24-h urine samples in breastfeeding women using Spearman’s rho.

*n* = 68		Urine	Urine Selenium	Urine Selenium	Urine Creatinine	Urine Creatinine	Selenium: Creatinine Ratio	Milk Selenium	Dietary Selenium
		Volume L	µg/L	µg/day	g/L	g/day	µg/g	µg/L	μg/day
Urine Selenium µg/L	*r*	−0.631							
	*p*	0.000							
Urine Selenium µg/day	*r*		0.642						
	*p*	ns	0.000						
Urine Creatinine g/L	*r*	−0.871	0.657						
	*p*	0.000	0.000	ns					
Urine Creatinine g/day	*r*								
	*p*	ns	ns	ns	ns				
Selenium Creatinine Ratio µg/g	*r*		0.580	0.879					
	*p*	ns	0.000	0.000	ns	ns			
Milk Selenium µg/day	*r*			0.269			0.280		
	*p*	ns	ns	0.000	ns	ns	0.025	ns	
Dietary Selenium µg/day	*r*				−0.357				
	*p*	ns	ns	ns	0.003	ns	ns	ns	
Total Energy Intake	*r*	0.247							0.405
	*p*	0.043	ns	ns	ns	ns	ns	ns	0.001
